# Development, Validation, and Utilization of a Social Media Use and Mental Health Questionnaire among Middle Eastern and Western Adults: A Pilot Study from the UAE

**DOI:** 10.3390/ijerph192316063

**Published:** 2022-11-30

**Authors:** Omar Hegazi, Samer Alalalmeh, Ahmad Alfaresi, Soheil Dashtinezhad, Ahmed Bahada, Moyad Shahwan, Ammar Abdulrahman Jairoun, Tesleem K. Babalola, Haya Yasin

**Affiliations:** 1 College of Pharmacy and Health Sciences, Ajman University, Ajman 346, United Arab Emirates; 2 College of Engineering, University of Sharjah, Sharjah 27272, United Arab Emirates; 3 Centre of Medical and Bio-allied Health Sciences Research, Ajman University, Ajman 346, United Arab Emirates; 4 Health and Safety Department, Dubai Municipality, Dubai 67, United Arab Emirates; 5 Program in Public Health, Renaissance School of Medicine, Stony Brook University, Stony Brook, NY 11794, USA

**Keywords:** stress, social media, mental health, young adults, questionnaire, validation

## Abstract

Objectives: We aimed to develop and validate a mental health stigma measurement tool for use within the social media context, utilizing the tool to assess whether the stigma shown in face-to-face interactions translates to social media, coupled with comparing whether social media use can cause the stigma among a sample of Middle Eastern and Western populations. Methods: The development and validation phase comprised a systematic process that was used to develop an assessment tool that could be used within the social media context and establish its validity and reliability. A 5-point Likert-type scale (1 = strongly disagree to 5 = strongly agree) was developed to assess mental health stigma. The anonymous questionnaire was distributed from June 2022 to August 2022 on various social media platforms and groups predominated by the two demographics of interest, enrolling 1328 participants (with only 1001 responses deemed valid). The utilization phase consisted of bivariate and multivariable analysis of the data. The cutoff points for low, medium, and high scores were the 25th, 50th, and 75th percentil, respectively. Results: The instrument comprised three dimensions: acceptance, intolerance, and digital care sentiment. In the Middle Eastern subset of participants, a higher score of intolerance (more stigma) toward mental illness was found in 72.4% of the participants, with a higher score of acceptance being 35.1% and of digital care sentiment being 46.4%. The mean scores for all the scales were as follows: intolerance (3.08 ± 0.64), acceptance (3.87 ± 0.71), and digital care sentiment (3.18 ± 0.69). For Westerners, a higher score of intolerance toward mental illness was found in 24.0% of the participants, with a higher score of acceptance being 56.8% and of digital care sentiment being 38.2%. The mean scores for all the scales were as follows: intolerance (2.28 ± 0.73), acceptance (4.21 ± 0.61), and digital care sentiment (3.08 ± 0.62). Various results were obtained regarding the effect of individual social media platforms on the different subscales. Conclusions: Stigma does follow people on social media, whether they are Middle Easterners or Westerners, although to varying degrees. The results of social media interaction and activity varied based on the group that used them, with some having an impact on one group but not the other. For these reasons, proper guidance is advised when utilizing and interacting with social media platforms.

## 1. Introduction

With the inception, and the inevitable growth, of social media, the way people communicate has drastically changed. The number of social media users worldwide was estimated to be 3.9 billion at the start of 2022 [1,2]. Ranging from blogs and micro-blogs (e.g., Twitter) and media-sharing sites (e.g., YouTube) to messaging (e.g., WhatsApp) and social networking sites (e.g., Facebook) [3]; social media, in public health and medicine, has been defined as conduits by which real-time and on-the-go communication is possible [4].

Manifesting in three ways—stereotype, prejudice, and discrimination [5]—and resulting in reduced autonomy and self-sufficiency [6], stigma is defined as a set of negative attitudes and values that, in most cases, motivate individuals to fear, avoid, and discriminate against people of a particular nature [5]. The mitigation of such a phenomenon within the mental health context, and its cause, has been a widely researched topic with varying results. Previous findings have concluded that more knowledge leads to less stigma [7,8], and contact with a person that is afflicted with a mental disorder can influence behaviors and attitudes [9]. Behaviors ranged from a positive and understanding attitude [10], thinking of them as people who need help [11], to negativity and rejection, making them prefer to keep a social distance under certain beliefs about how dangerous people with mental health disorders are [12,13,14,15,16].

Within the Arab countries’ context, and because they share a set of values and traditions that distinctly differ from those of the Western countries [17], mental illness is viewed from a spiritual perspective [18], deeming mental disorders the works of “evil” [18] or a trial from god that resulted from an act of sin [19]; making them view the condition negatively and consequently affecting their help-seeking behaviors in addition to expressing their psychological problems in the form of physical symptoms [20].

Social media platforms, and their effects, have piqued scientific interest since their widespread use among the populace. Previous findings highlighted positive aspects of social media as a tool to alleviate the burden of mental illness by allowing people suffering from such conditions to share their experiences, seek support from their peers, search for treatment information, and help them cope with symptoms [21,22,23]. Additional findings highlight that under proper guidance, social media use can lead to positive outcomes [24,25,26,27]. Different findings, however, link social media use with depression and anxiety [28], with more studies linking it to negative effects on mental health, health behaviors, social isolation, happiness, and mood [29,30,31,32,33,34,35]. A study by Robinson et al. showed that social media users seem to stigmatize and trivialize mental health conditions compared to physical ones [36]. It also highlights the potential for social media to measure public attitudes toward mental health conditions [36].

Studies on the impact of social media use on mental health have been conducted in certain Arabic countries such as Saudi Arabia and Lebanon, with the findings showing similar trends to those conducted worldwide regarding the association of increased screen time with depression and anxiety [37,38]. 

The recent outbreak of COVID-19 (SARS-CoV-2) and the isolation that came with the quarantine periods have undeniably impacted the time spent using social media platforms and caused a marked increase in the number of users [39,40,41,42,43]. The research conducted during recent years focused mostly on the effects of the COVID-19 pandemic, A study conducted by Geirdal et al. showed that those who reported increased and more frequent use of social media at the beginning of the pandemic experienced poorer psychological and mental health in countries such asthe United States and the United Kingdom [44]. Additional findings associate the surge of use with an increase in the likelihood of exhibiting symptoms of anxiety and depression [45]. An eight-year longitudinal study demonstrated a moderate link between the time spent using social networking sites, anxiety, and depression corroborating the displacement hypothesis. The same study, however, found no significant associations between time spent using social media and mental health across the length of the study [46].

At large, digital technological interventions have shown promise, with some illustrating the potential of digital interventions as a tool to break the barriers of conventional mental health services and the stigma that follows it [47], and others displaying its potential in furthering cognitive behavioral therapy [48]. Other findings similarly establish its effectiveness in the treatment of depressive disorders and anxiety disorders [49].

Although negative facets of social media use are present, positive aspects are apparent, some of the positive attributes include it facilitating social interaction [50], it allows instant contact between individuals with mental disorders and their care providers [51]. A recent review also underlined positive aspects of social media for the mentally ill, such as enhancing communication with family and friends, supplementing well-being and overall life satisfaction, causing greater independence and self-efficacy and it also showed the link between social media use and less depressive symptoms [52]. At present, an umbrella review conducted by Patti M. et al. highlighted some evidence found in the literature as well as the current research gaps [53].

To our knowledge, no studies compare the impact of social media platforms on Middle Eastern and Western young adults nor their sentiment on the use of digital technology in treating and assessing mental disorders. This study aimed to develop and validate a questionnaire to assess social media use and mental health among Middle Eastern and Western young adults. 

## 2. Methods

### 2.1. Development of the Questionnaire

This study was based on the Mental Health Knowledge Schedule (MAKS) [54], the Community Attitudes toward Mental Illness (CAMI) [55], and the Reported and Intended Behavior Scale (RIBS) [56]. The questionnaire was developed in the English language and was circulated to separate groups of undergraduates (n = 10) for input into the selection of items under each element, the phrasing of items, and feedback about the suitability of elements which resulted in consequent modifications. Subsequently, the questionnaire was revised and pilot-tested using another group of 30 people from different social media groups, following which further fine adjustments were made to produce the final version.

The questionnaire included twenty-nine unique questions and was in English. Of the 29 questions, a question not pertaining to any subscale was repeated twice at different intervals as an attentiveness check. Participants who gave the same answer to both questions were considered “attentive” and were included in the analysis, whilst those who gave different answers were considered “inattentive” and were excluded. The purpose of this attentiveness check is to ensure the participants’ full concentration and understanding of the questions. The first part of the questionnaire covered demographic characteristics, including social media platforms, age, gender, hours of social media use per day, and level of education. The level of education was divided into three basic categories because of the difference between education levels and curriculums in the targeted demographics. A five-point Likert-type scale was used to assess the different dimensions, ranging from strongly disagree to strongly agree: strongly disagree = 1, disagree = 2, neutral = 3, agree = 4, and strongly agree = 5.

### 2.2. Participants and Data Collection

Before this study was conducted, ethical approval was obtained from the Research Ethics Committee at Ajman University.

To ensure that the sample proportions would be within 5% of the ‘true’ population prevalences with a 95% level of confidence, 384 responses were required, this was achieved threefold.

Between June 2022 and August 2022, the questionnaire was distributed, through convenience sampling, to social media groups and communities across different platforms including Facebook, WhatsApp, Reddit, Twitter, LinkedIn, Telegram, and Discord. The sampling included distributing the questionnaire to pages and groups willing to participate in this study. These platforms were predominated by two main subsets; a Middle Eastern subset, and a Western subset. The Middle Eastern subset was observed to be dominated by people from Egypt, Jordan, Sudan, and the United Arab Emirates. The Westerner subset was observed to be predominated by people from the United States, Canada, the United Kingdom, Australia, and New Zealand.

During the data collection process, participants were provided with an explanatory statement that assured their anonymity and that participants had the right to accept or refuse participation in this study, with no financial incentive in exchange for participation. It also explained that participants had the right to withdraw at any point in this study. People less than the age of 18 years or those who did not indicate a willingness to participate were excluded from this he study.

### 2.3. Statistical Analysis

Statistical analysis took two distinct phases; the first was the questionnaire development and validation, starting with establishing what factors to use in the questionnaire. The second phase included bivariate and multivariable analysis, which was dubbed the utilization phase. All data were entered into Microsoft Excel, validated for accuracy, and analyzed using SPSS version 28 (IBM, Corp., Armonk, NY, USA). Descriptive statistics were computed for participant demographic information.

Before conducting any analysis, participants who failed the attentiveness check question were excluded. Factor analysis was conducted as the starting point in the questionnaire validation and was performed using Varimax rotation of factors with eigenvalues > 1. The item retention criteria were that the item’s factor loading should be higher than 0.30 and no higher loading on another factor [57]. Further characterization of items into “significant” and “very significant” was used based on the factor loadings, with loadings of 0.40 or greater considered the former and factor loadings ≥ 0.50 considered the latter.

Internal consistency was measured using Cronbach’s alpha to assess the internal consistency of each dimension in the scale, with values between 0.70 and 0.90 deemed acceptable [58]. The scores from each scale were determined by adding the scores for the individual items comprising the scale and dividing by the number of items to maintain a score range between 1 and 5. One-way repeated-measures ANOVA was performed to compare the respondents’ scores on the subscales.

In the utilization phase, the independent-sample t-test was used when comparing two means, whereas the ANOVA test was used to compare three means, with Fisher’s LSD post hoc test used to compare all pairwise differences. The point-biserial correlation was used between one continuous variable and one dichotomous variable [59,60], the dichotomous variable, in this case, being the use of a social media platform. Six hierarchical linear regressions were conducted in sets of three, with the main difference being the separation by region. In each set, the score of each subscale was used as the dependent variable and all sociodemographic factors and platforms that showed statistical significance (i.e., *p* < 0.05) in the bivariate analysis were taken as independent variables in the regression model to eliminate the capacity for confounding factors.

## 3. Results

### 3.1. Development and Validation

Overall, 1328 individuals completed the questionnaire, with 327 excluded due to failing the attentiveness check question. The data from 1001 participants were used in every analysis from this point onwards. The distribution of Middle Easterners (49.6%) and Westerners (50.4%) was almost equal, with more than half of the participants being females (61.0%), aged between 18 and 29 years old (61.1%), using social media for 1 to 5 h a day (59.4%). Participants’ demographics and other information are shown in Table 1. A summary of respondent answers is reported using descriptive statistics in Table S1.

The Kaiser–Meyer–Olkin (KMO) measure of sampling adequacy, which depicts the proportion of variance in variables influenced by the underlying factors, for the factor analysis was 0.86, with factor analysis extracting three distinct factors with a cumulative explained variance of 50%. Kaiser–Meyer–Olkin (KMO) values closer to 1.0 indicate that the conducted factor analysis may be useful. If the value is less than 0.50, the factor analysis results are unlikely to be meaningful [61]. Item rotation showed “very significant” (>0.50) loading of each item on one factor (Table 2), except for item 2, people with mental illness cannot take care of themselves and must be hospitalized, which was “significant” (>0.40). Subsequently, the 17 items were regrouped into three factors: intolerance, acceptance, and digital care sentiment. Items associated with each of the three factors are shown in Table 3 alongside the Cronbach’s alpha, which was >0.70 for all factors. The corrected item–scale correlations for all items met the criterion for retention and were >0.30; additionally, all were significant (*p* < 0.001). The results of the one-way repeated-measures ANOVA showed significant differences between the scales (*p* < 0.001).

### 3.2. Utilization

Based on the total scores of the intolerance, acceptance, and digital care sentiment subscales, the 25th, 50th, and 75th percentiles were used as cutoff points for low, medium, and high scores, respectively.

In the Middle Eastern subset of participants, a higher score of intolerance (more stigma) toward mental illness was found in 72.4% of the participants, with a higher score of acceptance being 35.1% and of digital care sentiment being 46.4%. Mean scores for all the scales were as follows: intolerance (3.08 ± 0.64), acceptance (3.87± 0.71), and digital care sentiment (3.18 ± 0.69).

While for Westerners, a higher score of intolerance toward mental illness was found in 24.0% of the participants, with a higher score of acceptance being 56.8% and of digital care sentiment being 38.2%. Mean scores for all the scales were as follows: intolerance (2.28 ± 0.73), acceptance (4.21 ± 0.61), and digital care sentiment (3.08 ± 0.62) (Table 4).

### 3.3. Bivariate Analysis

In the Middle Eastern subset of participants, the bivariate analysis of factors associated with intolerance (more stigma) showed a significantly higher mean score in males compared to females (3.23 vs. 2.95, *p* < 0.001), in those aged 30 years and above compared to those between 18 and 29 years (3.15 vs. 3.05, *p* = 0.045), in those that are not close to someone with a mental illness compared to being close to someone with a mental illness (3.18 vs. 2.92, *p* < 0.001), in those that used social media for 6 h or more per day compared to moderate use of 1 to 5 h a day (3.19 vs. 3.1, *p* = 0.03), and in those with no knowledge of online support groups compared to those who knew of it (3.24 vs. 2.97, *p* < 0.001).

A lower intolerance score was significantly associated with the use of Instagram (r = −0.139), LinkedIn (r = −0.210), YouTube (r = −0.110), WhatsApp (r = −0.127), and inactivity in any online community (r = −0.133).

Bivariate analysis taking the acceptance score (less stigma) as the dependent variable showed a significantly higher mean score in females compared to males (3.93 vs. 3.78, *p* = 0.009), in those aged between 18 and 29 years compared to those above 30 years (3.96 vs. 3.65, *p* < 0.001), in those familiar with a person with mental illness (4.15 vs. 3.67, *p* < 0.001), in those with knowledge of online support groups compared to those with no knowledge of it (4.02 vs. 3.62, *p* < 0.001), and in those with an Undergraduate degree compared to a Highschool degree (4.01 vs. 3.80, *p* = 0.006) and a Postgraduate degree (4.01 vs. 3.75, *p* = 0.001).

A higher acceptance score was significantly associated with the use of TikTok (r = 0.116), Snapchat (r = 0.092), WhatsApp (r = 0.169), and inactivity in any online community (r = 0.142), whilst Facebook (r = −0.164) was associated with a significant decrease in the acceptance score.

Bivariate analysis taking the digital care sentiment score as the dependent variable showed a significantly higher mean score in males compared to females (3.24 vs. 3.13, *p* = 0.034), in those with a Highschool degree compared to an Undergraduate degree (3.28 vs. 3.14, *p* = 0.044) and a Postgraduate degree (3.28 vs. 3.11, *p* = 0.028), and in those that used social media for 6 h or more per day compared to moderate use of 1 to 5 h a day (3.36 vs. 3.08, *p* < 0.001) and light use of less than an hour (3.36 vs. 3.09, *p* = 0.023).

A higher digital care sentiment score was significantly associated with the use of Discord (r = 0.134), activity in Facebook communities (r = 0.137), and activity in video game communities (r = 0.113), whilst the use of Pinterest (r = −0.112), WhatsApp (r = −0.099), in addition to not using any social media platform (r = −0.122), nor being active in an online community (r = −0.236) was associated with a significant decrease in the digital care sentiment score. 

In the Western subset of participants, the bivariate analysis of factors associated with intolerance showed a significantly higher mean score in Males compared to Females (2.42 vs. 2.21, *p* = 0.001), in those aged 30 years and above compared to those between 18 and 29 years (2.38 vs. 2.19, *p* = 0.002), in those that are not close to someone with a mental illness compared to being close to someone with a mental illness (2.52 vs. 2.19, *p* < 0.001), in those that used social media for 6 h or more per day compared to moderate use of 1 to 5 h a day (2.41 vs. 2.21, *p* = 0.017), and in those with no knowledge of online support groups compared to those who knew of it (2.40 vs. 2.26, *p* = 0.045).

A lower intolerance score was significantly associated with using Reddit (r = −0.216) and not being active in any online community (r = −0.098). A higher intolerance score was significantly associated with using LinkedIn (r = 0.110) and being involved in video game communities (r = 0.088).

The bivariate analysis taking the acceptance score as the dependent variable showed a significantly higher mean score in females compared to males (4.24 vs. 4.15, *p* = 0.046), in those aged between 18 and 29 years compared to those above 30 years (4.32 vs. 4.10, *p* < 0.001), in those familiar with a person with mental illness (4.33 vs. 3.91, *p* < 0.001), in those with knowledge of online support groups compared to those with no knowledge of it (4.26 vs. 3.98, *p* < 0.001), and in those that used social media for moderate use of 1 to 5 h a day compared to light use of less than an hour per day (4.28 vs. 4.05, *p* = 0.001).

A higher acceptance score was significantly associated with the use of Twitter (r = 0.108), Discord (r = 0.114), and Reddit (r = 0.233). 

The bivariate analysis taking the digital care sentiment score as the dependent variable showed a significantly higher mean score in females compared to males (3.12 vs. 3.01, *p* = 0.038), in those with knowledge of online support groups compared to those with no knowledge of it (3.11 vs. 2.96, *p* = 0.024), and in those that used social media for 6 h or more per day compared to moderate use of 1 to 5 h a day (3.27 vs. 3.05, *p* = 0.002) and light use of less than an hour (3.27 vs. 2.95, *p* < 0.001). 

A higher digital care sentiment score was significantly associated with the use of Pinterest (r = 0.093), TikTok (r = 0.107), Twitter (r = 0.183), Reddit (r = 0.097), and other platforms (r = 0.092), in addition to being active in Facebook communities (r = 0.122), and video game communities (r = 0.128), whilst WhatsApp (r = −0.104), in addition to not using any social media platform (r = −0.088), nor being active in an online community (r = −0.205) was associated with a significant decrease in the digital care sentiment scores (Table 5 and Table 6). 

### 3.4. Multivariable Analysis

In the Middle Eastern subset of participants, a first linear regression, taking the intolerance subscale as the dependent variable, showed that using social media for 6 h or more (Beta = 0.514) and being male (Beta = 0.546) were associated with higher intolerance towards mental illness, whereas using LinkedIn (Beta = −0.268) and YouTube (Beta = −0.165) were associated with lower intolerance toward mental illness. A second linear regression, taking the acceptance subscale as the dependent variable, showed that being familiar with people with mental illness (Beta = 0.761) and having knowledge of online support groups (Beta = 0.755) were associated with higher acceptance towards mental illness. A third linear regression, taking the digital care sentiment scale as the dependent variable, showed that more stigmatizing attitudes (higher intolerance score) (Beta = 0.470) and extensive use of social media (0.457) were associated with higher favorable behaviors towards the move to digital mental health care.

In the Western subset of participants, A first linear regression, taking the intolerance subscale as the dependent variable, showed that being male (Beta = 0.464) and being 30 years old and above (Beta = 0.380) were associated with higher intolerance toward mental illness, whereas using Reddit (Beta = −0.432) was associated with lower intolerance toward mental illness. A second linear regression, taking the acceptance subscale as the dependent variable, showed that being familiar with people with mental illness (Beta = 0.761) and having knowledge of online support groups (Beta = 0.769) were associated with higher acceptance towards mental illness. A third linear regression, taking the digital care sentiment subscale as the dependent variable, showed that more stigmatizing attitudes (higher intolerance score) (Beta = 0.382) and having knowledge of online support groups (Beta = 0.695) were associated with higher favorable behaviors towards the move to digital mental health care. (Table 7 and Table 8).

## 4. Discussion

To the best of our knowledge, this is the first study that compares and assesses attitudes and behaviors toward mentally ill people in a social media setting between Middle Easterners and Westerners, as well as assessing their digital care sentiment.

The results align with other studies, albeit in a different context, with stigma being a prevalent phenomenon in many Middle Eastern societies [62,63,64,65]. Such stigma can be seen in Dardas’s paper, where Arabs consider mentally ill people a shame [17], and in Dalky’s findings, where Arabs believed it to be a disgrace to the family’s reputation to care for a family member with a mental illness [66]. With families holding such negative attitudes towards the mentally ill, and care of such individuals falling in the hands of said family members [66], it takes families extended periods, that can reach years in time, to accept that their mentally ill family member requires professional psychiatric care [17].

There is limited previous literature examining area influences on mental health stigma, let alone the effect of social media on mental health across different regions. In our study, the stigma observed in the Western subset of participants was below that of its Middle Eastern counterpart, this can be attributed to the many anti-stigma activities [67] and campaigns (e.g., the Global Anti-Stigma Alliance) [68] that can be found thorough the region. However, in a vacuum, Western individuals with mental health disorders do still face stigma. A study conducted on the French public by Angermeyer et al. showed misconceptions about mental disorders in the population [69]. Another study conducted by the United Kingdom Department of Health showed that, in comparison to Scotland, England established a more significant negative trend in mental health-related attitudes [70].

The association between age, knowledge, and mental illness is still controversial. In both subsets of our findings, older people showed more stigma, which is consistent with those reported in a Lebanese national study [71] but opposes the results of other papers that associate age with bounds of experience, said experience leads to more knowledge, hence lowering their stigmatic views [72]. Our findings were in accordance with other studies regarding the effects of gender, showing that females had a better attitude towards the mentally ill [73]; this can be attributed to them being more empathetic and tolerant [74,75]. The effect of education on Middle Easterners’ stigma in our study is consistent with other studies, showing that higher knowledge leads to less stigma [7,8,71,76]; however, our findings showed that education did not affect Westerners. In our findings, longer hours of social media use were associated with increased stigma. 

Familiarity with people suffering from a mental illness and online support groups were associated with more positive attitudes in our findings. Studies have shown that experience with people that have mental disorders has been associated with more benevolence and positive attitudes [71,74]. While others illustrated the negative attitude exhibited by those close to someone with a mental illness [77].

### 4.1. Platforms and Activity

Stigma towards individuals with mental health issues on social media has been observed in many studies [36,78,79,80], with some of them indicating that the use of specific social media platforms such as Facebook is associated with different mental disorders [81] or Instagram, for instance, which was associated with body image and self-harm issues [82]. In our study, the use of different platforms had varying results depending on whether the user was of Middle Eastern origin or Western origin.

In the Middle Eastern subset, the use of Instagram, LinkedIn, YouTube, and WhatsApp, in addition to not being active in any online community, was associated with lower stigma. Acceptance of the mentally ill was associated with using TikTok, Snapchat, and WhatsApp, in addition to not being active in any online community. These results align with other findings about social media being used as a tool that allows people to share their experiences and seek support from their peers [21,22,23]. However, the use of Facebook was associated with a decrease in such acceptance, which aligns with papers that highlight the negative relationship between social media and mental health [83,84,85]. 

In the Western subset, the use of Reddit and not being active in any online community was associated with lower stigma. However, using LinkedIn and being involved in video game communities was associated with increased stigma. The mentally ill’s acceptance was associated with using Twitter, Discord, and Reddit. Other studies suggest a positive relationship between Twitter and mental health, which aligns with our findings [86,87,88,89].

In summary, most of the observed platforms had varying effects depending on the subset that used them, with some affecting one group and not another (e.g., Reddit) and others exhibiting opposite effects (LinkedIn).

The higher sentiment towards the move to digital care in Middle Eastern participants can be due to many factors. For instance, a study conducted by Al-Krenawiin in 2005 found that Arab patients with mental illness avoid the adverse reactions of those around them by not disclosing their symptoms to others [20].

### 4.2. Theoretical and Practical Contributions

Our study contributes to the relevant literature in various ways. First, this study attempts to directly evaluate the attitudes of Middle Easterners and Westerners toward the mentally ill online. Secondly, it advances our understanding of the drastic differences between the different subsets of participants (mainly Middle Easterners and Westerners), which is a step towards finding a solution to the stigma that is found in the digital world. Thirdly, it attempts to decern individual factors and their effects on said attitudes (sociodemographic factors, platforms, and activity) and find a causal relationship between said factors and individual attitudes using statistical approaches. 

### 4.3. Limitations and Strengths

This study used a relatively large sample, which included measures specifically targeted at evaluating stigma in social media. It provides a first-time comparison between the effects of different platforms on both Middle Easterners’ and Westerners’ mental health stigma. Although the total sample could be considered significant, 500 participants in each group is small. This study is cross-sectional with a low level of evidence. The instruments used in the assessment are newly developed and need further validation in each context. An information bias could exist due to the data being self-reported. The results could not be generalized to the entire population since some groups hold much of the sample. Future research using alternative methods might obtain a complete view of the level of effects.

## 5. Conclusions

Our main findings were that stigma does follow people on social media, whether they are Middle Easterners or Westerners, although to varying degrees. Social media activity and platforms had varying effects depending on the group that used them, with some affecting one group and not another (e.g., Reddit) and others exhibiting opposite effects (LinkedIn), which is why proper guidance is needed when using and interacting with social media platforms.

## Figures and Tables

**Table 1:** Respondents’ demographics and other information.

Variables	N (%)
**Origin**	
The Middle East and North Africa (MENA)	496 (49.6)
Westerners	505 (50.4)
**Gender**	
Female	611 (61.0)
Male	390 (39.0)
**Age**	
18 to 29 years old	612 (61.1)
30+ years old	389 (38.9)
**Highest level of education**	
Highschool	264 (26.4)
Undergraduate degree	384 (38.4)
Postgraduate degree	353 (35.3)
**Social media use in a day**	
Light use (less than an hour)	136 (13.6)
Moderate use (1 to 5 h)	595 (59.4)
Severe use (6+ h)	270 (27)
**An active member in which communities?** ^a^	
Active Facebook communities	244 (20.7)
Active in video games communities	161 (13.7)
Other communities	260 (22.1)
Not active on any online community	511 (43.5)
**Have you heard of online support groups?**	
No	277 (27.7)
Yes	724 (72.3)
**Does a relative, friend, or anyone you know suffer from a mental illness?**	
No	438 (43.8)
Yes	563 (56.2)
**Social media platforms used ^a^**	
Facebook	425 (12)
Instagram	648 (18.3)
LinkedIn	262 (7.4)
Pinterest	128 (3.6)
TikTok	315 (8.9)
Twitter	228 (6.4)
Snapchat	240 (6.8)
YouTube	547 (15.5)
Discord	115 (3.3)
Reddit	163 (4.6)
WhatsApp	406 (11.5)
Other	40 (1.1)
None	20 (0.6)
**How many people do you think are underdiagnosed with depression? ^b^**	
6%	62 (6.2)
11%	132 (13.2)
20%	336 (33.6)
35%	471 (47.1)

^a^ Respondents were allowed to choose multiple platforms and communities. ^b^ Attentiveness check question.

**Table 2:** Rotated factor loadings for current items.

Item	Factor ^a^		
	1	2	3
Mental illness is a state of mind and not a physical condition.	*	0.721	*
2. People with mental illness cannot take care of themselves and must be hospitalized.	*	0.458	*
3. There is something about mentally ill people’s behavior online that makes it easy to tell them from ordinary people.	*	0.756	*
4. People who develop signs of mental disorders should be limited from using social media or outright forbidden.	*	0.613	*
5. Mentally ill people are hostile or aggressive.	*	0..505	*
6. Anyone with a history of mental disorders should be excluded from having any role with authority over others (e.g., admins) in an online community.	*	0.520	*
7. People develop mental disorders due to heavier, emotional interactions with online communities and social media.	*	0.699	*
8. Mental disorders are health conditions like any other.	0.545	*	*
9. I would continue being an online friend to someone after discovering their mental disorder.	0.761	*	*
10. I would be willing to engage in a relationship with someone that has a controlled mental disorder.	0.778	*	*
11. Mentally ill people can live normally within a community.	0.705	*	*
12. An online support community or an online therapist would be safer for people in an actual community.	*	*	0.570
13. Communities on social media can have a therapy-like effect on people with mental illness.	*	*	0.690
14. Online support groups have a meaningful impact on one’s mental health.	*	*	0.740
15. An online therapist or an online support group would be more convenient for my confidentiality.	*	*	0.627
16. I would be comfortable sharing personal stories with members of an online support group.	*	*	0.704
17. Online therapists can replace face-to-face interaction with therapists or counselors.	*	*	0.599

^a^ Loadings < 0.30.a. 1 = acceptance, 2 = intolerance, 3 = digital care sentiment.

**Table 3:** Extracted three factors with corresponding items.

Factors and Items	Item Mean	Factor Mean	SD	Alpha
**Intolerance**		2.68	0.79	0.81
*Mental illness is a state of mind and not a physical condition.*	3.09			
*People with mental illness cannot take care of themselves and must be hospitalized.*	2.52			
*There is something about mentally ill people’s behavior online that makes it easy to tell them from ordinary people.*	2.80			
*People who develop signs of mental disorders should be limited from using social media or outright forbidden.*	2.45			
*Mentally ill people are hostile or aggressive.*	2.40			
*Anyone with a history of mental disorders should be excluded from having any role with authority over others (e.g., admins) in an online community.*	2.36			
*People develop mental disorders due to heavier, emotional interactions with online communities and social media.*	3.10			
**Acceptance**		4.04	0.68	0.71
*Mental disorders are health conditions like any other.*	4.13			
*I would continue being an online friend to someone after discovering their mental disorder.*	4.15			
*I would be willing to engage in a relationship with someone that has a controlled mental disorder.*	3.85			
*Mentally ill people can live normally within a community.*	4.03			
**Digital care sentiment**		3.23	0.61	0.74
*An online support community or an online therapist would be safer for people in an actual community.*	3.05			
*Communities on social media can have a therapy-like effect on people with mental illness.*	3.28			
*Online support groups have a meaningful impact on one’s mental health.*	3.60			
*An online therapist or an online support group would be more convenient for my confidentiality.*	3.12			
*I would be comfortable sharing personal stories with members of an online support group.*	3.06			
*Online therapists can replace face-to-face interaction with therapists or counselors.*	2.68			

**Table 4:** Scores of each dimension across the Middle Eastern and Western subsets.

Variable	N (%)		Mean Scores ± SD	
	MENA	WE	MENA	WE
**Intolerance**			3.08 ± 0.64	2.28 ± 0.73
Low	41 (8.3)	229 (45.3)		
Medium	96 (19.4)	155 (30.7)		
High	359 (72.4)	121 (24.0)		
**Acceptance**			3.87± 0.71	4.21 ± 0.61
Low	178 (35.9)	79 (15.6)		
Medium	144 (29.0)	139 (27.5)		
High	174 (35.1)	287 (56.8)		
**Digital care sentiment**			3.18 ± 0.69	3.08 ± 0.62
Low	158 (31.9)	184 (36.4)		
Medium	108 (21.8)	128 (25.3)		
High	230 (46.4)	193 (38.2)		

Middle East and North Africa (MENA), Westerners (WE).

**Table 5:** Bivariate analysis of sociodemographic factors associated with each subscale.

Variable	Intolerance		Acceptance		Digital Care Sentiment	
	MENA	WE	MENA	WE	MENA	WE
**Gender**						
Male	3.23 ± 0.57	2.42 ± 0.72	3.78 ± 0.64	4.15 ± 0.55	3.24 ± 0.67	3.01 ± 0.66
Female	2.95 ± 0.66	2.21 ± 0.72	3.93 ± 0.77	4.24 ± 0.63	3.13 ± 0.70	3.12 ± 0.59
*p*-value	**<0.001**	**0.001**	**0.009**	**0.046**	**0.034**	**0.038**
**Age categories**						
18–29 years old	3.05 ± 0.64	2.19 ± 0.70	3.96 ± 0.72	4.32 ± 0.59	3.19 ± 0.71	3.09 ± 0.56
30+ years old	3.15 ± 0.62	2.38 ± 0.75	3.65 ± 0.67	4.10 ± 0.60	3.15 ± 0.64	3.07 ± 0.67
*p*-value	**0.045**	**0.002**	**<0.001**	**<0.001**	0.290	0.374
**Education level**						
Highschool	3.15 ± 0.54	2.25 ± 0.69	3.80 ± 0.72	4.23 ± 0.70	3.28 ± 0.64	3.10 ± 0.64
Undergraduate degree	3.07 ± 0.63	2.30 ± 0.73	4.01 ± 0.72	4.19 ± 0.61	3.14 ± 0.73	3.07 ± 0.61
Postgraduate degree	3.01 ± 0.74	2.28 ± 0.75	3.75 ± 0.66	4.23 ± 0.55	3.11 ± 0.68	3.09 ± 0.62
*p*-value	0.145	0.843	**0.002**	0.761	**0.052**	0.882
**Hours of use**						
Light use	3.06 ± 0.62	2.36 ± 0.76	3.71 ± 0.65	4.05 ± 0.60	3.09 ± 0.60	2.95 ± 0.74
Moderate use	3.01 ± 0.65	2.21 ± 0.70	3.92 ± 0.75	4.28 ± 0.59	3.08 ± 0.73	3.05 ± 0.56
Severe use	3.19 ± 0.59	2.41 ± 0.77	3.81 ± 0.66	4.16 ± 0.63	3.36 ± 0.60	3.27 ± 0.57
*p*-value	**0.013**	**0.028**	0.103	**0.003**	**<0.001**	**<0.001**
**Familiarity with online support groups**						
No	3.24 ± 0.53	2.40 ± 0.79	3.62 ± 0.68	3.98 ± 0.67	3.22 ± 0.64	2.96 ± 0.70
Yes	2.97 ± 0.68	2.26 ± 0.72	4.02 ± 0.69	4.26 ± 0.58	3.15 ± 0.72	3.11 ± 0.60
*p*-value	**<0.001**	**0.045**	**<0.001**	**<0.001**	0.142	**0.024**
**Familiarity with someone with a mental illness**						
No	3.18 ± 0.58	2.52 ± 0.69	3.67 ± 0.68	3.91 ± 0.66	3.21 ± 0.66	3.08 ± 0.64
Yes	2.92 ± 0.68	2.19 ± 0.72	4.15 ± 0.67	4.33 ± 0.54	3.13 ± 0.74	3.08 ± 0.61
*p*-value	**<0.001**	**<0.001**	**<0.001**	**<0.001**	0.126	0.447

Middle East and North Africa (MENA), Westerners (WE). Higher scores on “intolerance” signify greater amounts of stigma, while higher scores on “acceptance” signifies lower stigma and a higher score on “digital care sentiment” signifies higher acceptance towards the move to online mental health care. The bold is for the values that are statically significant (*p* < 0.05).

**Table 6:** Correlation between platforms, activity, and subscales.

Variable		Intolerance		Acceptance		Digital Care Sentiment	
		MENA	WE	MENA	WE	MENA	WE
Active Facebook communities	r	0.066	0.068	−0.080	−0.007	0.137	0.122
	*p*-value	0.141	0.124	0.076	0.874	**0.002**	**0.006**
Active video games communities	r	0.049	0.088	−0.025	0.009	0.113	0.128
	*p*-value	0.279	**0.048**	0.580	0.836	**0.012**	**0.004**
Not active on any online community	r	−0.133	−0.098	0.142	0.042	−0.236	−0.205
	*p*-value	**0.003**	**0.028**	**0.001**	0.347	**<0.001**	**<0.001**
Facebook	r	0.035	−0.031	−0.164	0.013	0.037	0.055
	*p*-value	0.438	0.485	**<0.001**	0.762	0.410	0.221
Instagram	r	−0.139	0.063	0.082	0.019	0.004	0.071
	*p*-value	**0.002**	0.158	0.067	0.677	0.929	0.111
LinkedIn	r	−0.210	0.110	−0.015	−0.048	−0.041	0.019
	*p*-value	**<0.001**	**0.013**	0.744	0.284	0.365	0.675
Pinterest	r	−0.069	0.000	−0.024	0.051	−0.112	0.093
	*p*-value	0.124	0.994	0.587	0.250	**0.012**	**0.037**
TikTok	r	0.035	0.036	0.116	0.055	0.023	0.107
	*p*-value	0.442	0.423	**0.009**	0.214	0.608	**0.017**
Twitter	r	−0.007	0.007	0.007	0.108	0.007	0.183
	*p*-value	0.872	0.874	0.876	**0.015**	0.880	**<0.001**
Snapchat	r	−0.003	−0.014	0.092	0.066	0.020	−0.022
	*p*-value	0.946	0.760	**0.040**	0.137	0.657	0.617
YouTube	r	−0.110	0.070	0.057	−0.005	0.046	−0.005
	*p*-value	**0.014**	0.118	0.204	0.903	0.302	0.904
Discord	r	−0.020	−0.044	0.048	0.114	0.134	0.087
	*p*-value	0.660	0.323	0.283	**0.010**	**0.003**	0.051
Reddit	r	−0.081	−0.216	0.060	0.233	0.078	0.097
	*p*-value	0.071	**<0.001**	0.182	**<0.001**	0.081	**0.029**
WhatsApp	r	−0.127	0.017	0.169	0.001	−0.099	−0.104
	*p*-value	**0.004**	0.705	**<0.001**	0.981	**0.028**	**0.019**
Other	r	−0.030	−0.047	−0.043	0.030	−0.002	0.092
	*p*-value	0.512	0.288	0.337	0.501	0.972	**0.038**
None	r	0.042	−0.029	−0.032	−0.081	−0.122	−0.088
	*p*-value	0.350	0.510	0.477	0.068	**0.006**	**0.049**

Middle East and North Africa (MENA); Westerner (WE). The bold is for the values that are statically significant (*p* < 0.05).

**Table 7:** Multivariable analysis of the Middle Eastern subset.

Model 1: Linear Regression Taking the Intolerance Subscale as the Dependent Variable.					
	Unstandardized Beta	Standardized Beta	*p*-Value	Confidence Interval	
				Lower Bound	Upper Bound
Males Compared to females	0.546	0.267	<0.001	0.431	0.660
Age 30 years and above compared to 18–29 years	0.501	0.219	<0.001	0.383	0.620
6+ h of social media use compared with 1 to 5 h	0.514	0.385	<0.001	0.434	0.595
Knowledge of online support groups (no vs. yes)	0.213	0.114	<0.001	0.104	0.323
Familiarity with people with mental illness (no vs. yes)	0.148	0.070	0.011	0.034	0.262
Using LinkedIn compared to not using LinkedIn	−0.268	−0.034	0.002	−0.434	−0.102
Using YouTube compared to not using YouTube	−0.165	−0.037	0.010	−0.290	−0.040
Variables entered: gender, age, hours of social media use, knowledge of online support groups, familiarity with people who have a mental illness, and the use of LinkedIn, and YouTube.					
**Model 2: Linear Regression Taking the Acceptance Subscale as the Dependent Variable.**					
	**Unstandardized Beta**	**Standardized Beta**	***p*-Value**	**Confidence interval**	
				**Lower Bound**	**Upper Bound**
Age 30 years and above compared to 18–29 years	0.239	0.084	0.002	0.090	0.388
Males Compared to females	0.505	0.197	<0.001	0.385	0.624
Education level	0.123	0.066	0.010	0.030	0.216
Knowledge of online support groups (yes vs. no)	0.755	0.323	<0.001	0.633	0.877
Familiarity with people with mental illness (yes vs. no)	0.761	0.288	<0.001	0.632	0.891
Using TikTok compared to not using TikTok	0.234	0.035	0.002	0.083	0.386
Using Snapchat compared to not using Snapchat	0.280	0.037	0.001	0.119	0.441
Using WhatsApp compared to not using WhatsApp	0.272	0.038	<0.001	0.122	0.423
Variables entered: gender, age, education level, knowledge of online support groups, familiarity with people who have a mental illness, the use of TikTok, Snapchat, and WhatsApp.					
**Model 3: Linear Regression Taking the Digital Care Sentiment Subscale as the Dependent Variable.**					
	**Unstandardized Beta**	**Standardized Beta**	***p*-Value**	**Confidence Interval**	
				**Lower Bound**	**Upper Bound**
Males Compared to females	0.184	0.087	0.006	0.052	0.316
Education level	0.169	0.110	<0.001	0.096	0.243
Hours of social media use	0.457	0.330	<0.001	0.364	0.550
Active on Facebook communities compared to inactivity on Facebook communities	0.196	0.023	0.035	0.013	0.378
Higher Intolerance score (Intolerant attitudes)	0.470	0.454	<0.001	0.382	0.557
Using Discord compared to not using Discord	0.225	0.023	0.039	0.011	0.439
Variables entered: gender, education level, hours of social media use, being active on Facebook communities, intolerance score, and the use of Discord.					

**Table 8:** Multivariable analysis of the Westerner subset.

Model 1: Linear Regression Taking the Intolerance Subscale as the Dependent Variable.					
	Unstandardized Beta	Standardized Beta	*p*-Value	Confidence Interval	
				Lower Bound	Upper Bound
Age 30 years and above compared to 18–29 years	0.380	0.248	<0.001	0.263	0.496
Males Compared to females	0.464	0.273	<0.001	0.339	0.589
Hours of social media use	0.322	0.282	<0.001	0.232	0.411
Knowledge of online support groups (no vs. yes)	0.254	0.199	<0.001	0.123	0.385
Using LinkedIn compared to not using LinkedIn	0.198	0.050	0.003	0.067	0.329
Using Reddit compared to not using Reddit	−0.432	−0.090	<0.001	−0.583	−0.281
Variables entered: gender, age, hours of social media use, knowledge of online support groups, and the use of LinkedIn, and Reddit.					
**Model 2: Linear Regression Taking the Acceptance Subscale as the Dependent Variable.**					
	**Unstandardized Beta**	**Standardized Beta**	***p*** **-Value**	**Confidence interval**	
				**Lower Bound**	**Upper Bound**
Age 30 years and above compared to 18–29 years	0.194	0.071	0.001	0.080	0.307
Males Compared to females	0.321	0.106	<0.001	0.200	0.442
Hours of social media use	0.347	0.171	<0.001	0.259	0.435
Knowledge of online support groups (yes vs. no)	0.769	0.338	<0.001	0.625	0.913
Familiarity with people with mental illness (yes vs. no)	0.761	0.318	<0.001	0.634	0.887
Using Reddit compared to not using Reddit	0.147	0.017	0.049	0.001	0.293
Variables entered: gender, age, hours of social media use, knowledge of online support groups, familiarity with people who have a mental illness, and the use of Reddit.					
**Model 3: Linear Regression Taking the Digital Care Sentiment Subscale as the Dependent Variable.**					
	**Unstandardized Beta**	**Standardized Beta**	***p*** **-Value**	**Confidence interval**	
				**Lower Bound**	**Upper Bound**
Males Compared to females	0.123	0.055	0.032	0.011	0.235
Hours of social media use	0.349	0.233	<0.001	0.268	0.431
Knowledge of online support groups (yes vs. no)	0.695	0.415	<0.001	0.586	0.803
Active on Facebook communities compared to inactivity on Facebook communities	0.103	0.019	0.094	−0.017	0.224
Intolerance scores	0.382	0.291	<0.001	0.309	0.454
Variables entered: gender, hours of social media use, knowledge of online support groups, being active on Facebook communities, and intolerance score.					

## Data Availability

The data is contained within the article and Supplementary Material. Further data is available from the corresponding author upon reasonable request after approval from all the authors.

## Supplementary Materials

The following supporting information can be downloaded at: https://www.mdpi.com/article/10.3390/ijerph192316063/s1, Table S1: Summary of respondent answers (DOCX).
