# Supplementary material for: Development, Validation, and Utilization of a Social Media Use and Mental Health Questionnaire among Middle Eastern and Western Adults: A Pilot Study from the UAE
Source: Int J Environ Res Public Health. 2022 Nov 30;19(23):16063. doi: 10.3390/ijerph192316063 (PMC9736958; doi:10.3390/ijerph192316063)
Supplement: Supplementary file 1 [file ijerph-19-16063-s001.zip › ijerph-2005919-supplementary.pdf]

Item	Strongly disagree N (%)	Disagree N (%)	Neutral N (%)	Agree N (%)	Strongly Agree N (%)	Mean ± SD
1. Mental illness is a state of mind and not a physical condition	163 (16.3)	204 (20.4)	190 (19)	270 (27)	174 (17.4)	3.09 ± 1.35
2. People with mental illness cannot take care of themselves and must be hospitalized	206 (20.6)	311 (31.1)	287 (28.7)	147 (14.7)	50 (5.0)	2.52 ± 1.12
3. There is something about mentally ill people's behavior online that makes it easy to tell them from ordinary people	171 (17.1)	267 (26.7)	242 (24.2)	231 (23.1)	90 (9.0)	2.80 ± 1.23
4. People who develop signs of mental disorders should be limited from using social media or outright forbidden	255 (25.5)	314 (31.4)	218 (21.8)	157 (15.7)	57 (5.7)	2.45 ±1.19
5. Mentally ill people are hostile or aggressive	187 (18.7)	374 (37.4)	314 (31.4)	105 (10.5)	21 (2.1)	2.40 ± 0.97
6. Anyone with a history of mental disorders should be excluded from having any role with authority over others (e.g., admins) in an online community	276 (27.6)	323 (32.3)	209 (20.9)	150 (15)	43 (4.3)	2.36 ± 1.16
7. People develop mental disorders due to heavier, emotional interactions with online communities and social media	74 (7.4)	204 (20.4)	348 (34.8)	295 (29.5)	80 (8)	3.10 ±1.05
8. Mental disorders are health conditions like any other	25 (2.5)	69 (6.9)	109 (10.9)	346 (34.6)	452 (45.2)	4.13 ± 1.02
9. I would continue being an online friend to someone after discovering their mental disorder	11 (1.1)	32 (3.2)	131 (13.1)	445 (44.5)	382 (38.2)	4.15 ± 0.85
10. I would be willing to engage in a relationship with	18 (1.8)	77 (7.7)	220 (22)	407 (40.7)	279 (27.9)	3.85 ± 0.97

someone that has a controlled mental disorder						
11. Mentally ill people can live normally within a community	11 (1.1)	58 (5.8)	162 (16.2)	432 (43.2)	338 (33.8)	4.03 ± 0.91
12. An online support community or an online therapist would be safer for people in an actual community	45 (4.5)	209 (20.9)	458 (45.8)	228 (22.8)	61 (6.1)	3.05 ±0.93
13. Communities on social media have a therapy-like effect on people with mental illness	35 (3.5)	139 (13.9)	407 (40.7)	354 (35.4)	66 (6.6)	3.28 ± 0.91
14. Online support groups have a meaningful impact on one's mental health	14 (1.4)	78 (7.8)	306 (30.6)	504 (50.3)	99 (9.9)	3.60 ± 0.82
15. An online therapist or an online support group would be more convenient for my confidentiality	64 (6.4)	225 (22.5)	334 (33.4)	285 (28.5)	93 (9.3)	3.12 ± 1.06
16. I would be comfortable sharing personal stories with members of an online support group	81 (8.1)	251 (25.1)	274 (27.4)	317 (31.7)	78 (7.8)	3.06 ± 1.10
17. Online therapists can replace face-to-face interaction with therapists or counselors	153 (15.3)	320 (32)	271 (27.1)	210 (21)	47 (4.7)	2.68 ± 1.11
